# Hierarchical Whole-Body Control for Tendon-Cable-Driven Humanoids via Reference-Residual Policy and Offline-Learned Tendon Mapping

**DOI:** 10.3390/biomimetics11090607

**Published:** 2026-08-26

**Authors:** Wencong Gan, Jiehui Chen, Qingdu Li, Haiming Mou, Jianwei Zhang

**Affiliations:** 1 School of Optical-Electrical and Computer Engineering, University of Shanghai for Science and Technology, Shanghai 200093, China; 241260274@st.usst.edu.cn (W.G.);; 2 Institute of Machine Intelligence, University of Shanghai for Science and Technology, Shanghai 200093, China; 3 Shanghai DroidUp Co., Ltd., Shanghai 200433, China; 4 Department of Informatics, University of Hamburg, 20146 Hamburg, Germany

**Keywords:** full-size humanoid robot, bionic tendon-cable drive, whole-body control, reinforcement learning, multi-source motion library

## Abstract

Tendon-cable transmission can reduce distal-limb inertia in full-size humanoids, but its elasticity, hysteresis, backlash, and multi-joint coupling introduce state-dependent joint-to-motor discrepancies. We present a hierarchical whole-body tracking framework for the 28-DoF Droid X3 that separates high-level motion learning from transmission compensation. A reference-residual policy is trained in simulation by single-stage proximal policy optimization (PPO) using a unified robot-space motion representation, globally anchored tracking rewards, hierarchical hard-example sampling, and tendon-oriented domain randomization. In simulation checkpoint evaluation, more than 90% of 12,674 tested reference motions are completed. Independently, a state-conditioned mapper is trained offline through a differentiable motor–joint forward model identified from physical motor-excitation data and connected in series between the frozen policy and the low-level motor controller. Randomized repeated Mapping-OFF/ON trials are conducted on two nominally identical Droid X3 units. Within every robot–motion block, the frozen PPO checkpoint, reference trajectory, controller settings, safety bounds, and frozen mapper weights are held fixed; complete trials are the statistical units. OFF converts desired joint positions with the robot-specific fixed static calibration, whereas ON feeds the complete policy-level desired-joint vector and measured plant state to the frozen mapper, which directly outputs the complete motor-position command. Across the complete physical trials, the aggregate action-completion rate is 68% with Mapping OFF and 79% with Mapping ON, an increase of 11 percentage points. Representative walk, squat, and dance trajectories illustrate lower tracking errors under Mapping ON, while individual frames and selected temporal fragments are used only for visualization.

## 1. Introduction

Humanoid robots can operate in human-built environments without requiring infrastructure designed for specialized machines. Tendon-cable-driven architectures additionally place part of the actuation away from the limb extremities and introduce compliant transmission. In this paper, “lightweight” is used only in a platform-specific sense: Droid X3 has a total mass of approximately 28 kg, and its remote-transmission layout is intended to reduce distal-limb mass and inertia. No controlled mass or segment-inertia comparison is reported, and compliance is not treated as evidence of certified contact safety. Cable elasticity, pulley friction, backlash, hysteresis, and multi-joint coupling make the actuator-to-joint relation state-dependent and nonlinear. Combined with an underactuated floating base and intermittent foot contact, these effects complicate accurate whole-body tracking.

Reference-motion tracking has become a central paradigm for humanoid control. Here, it denotes a feedback policy conditioned on a time-indexed target trajectory and trained to reduce errors in joint configuration, body-link pose, velocity, and global-anchor state, while preserving balance and feasible contacts. It is distinct from open-loop joint playback, unconditional motion generation, motion planning, and upstream human-pose estimation. DeepMimic [1] and subsequent studies [2,3,4,5,6,7,8,9,10,11,12,13,14,15,16,17,18,19,20,21,22,23] extend this formulation to large motion libraries, teleoperation interfaces, and heterogeneous motion sources.

The literature nevertheless leaves several gaps that are especially relevant to the platform studied here. Some teleoperation systems separate upper- and lower-body objectives or introduce specialized modules to preserve stability [9,10,11]; long-horizon systems explicitly introduce localization feedback because open-loop tracking can accumulate position and heading drift [20,21]. Multi-source motion studies further show that skeleton definition, retargeting quality, sampling rate, and source-specific noise affect tracking feasibility and generalization [15,16,22]. Finally, most general motion trackers are evaluated on rigidly actuated humanoids, whereas full-size tendon-cable transmission introduces state-dependent actuator-to-joint errors that are not represented by an ideal independent-joint interface. Our scope is this last transmission-aware tracking problem; we do not claim to establish outdoor navigation, standardized human-contact safety, or general human–robot interaction performance.

This paper focuses on the Droid X3 experimental unit, a full-size tendon-cable-driven bipedal humanoid developed by Shanghai DroidUp Co., Ltd. [24]. Its remote-transmission layout is intended to reduce distal-limb inertia, but this mechanical motivation is not treated as a measured safety result. The mapping between joint-side motion and actuator-side output is nonlinear and affected by cable elastic deformation, friction, backlash, hysteresis, tension variation, and parameter drift; multi-joint tendon coupling may further cause one actuation chain to influence several joints, or several chains to jointly determine one joint’s response. For a bipedal system, these transmission uncertainties interact with the underactuated floating base, foot–ground contact switching, and whole-body center-of-mass regulation, making accurate joint-space execution difficult.

We therefore propose a hierarchical reference-residual tracking framework for Droid X3. The high-level actor receives proprioceptive history and a unified reference command and produces a bounded residual around the reference joint target. It is optimized directly by single-stage PPO without a subsequent teacher–student distillation stage. A separate state-conditioned mapper is trained offline from physical motor–joint excitation data. At deployment, the frozen mapper uses the desired joint position and measured joint/motor state to output a bounded motor-position command. The robot’s original fixed static calibration is retained as the Mapping-OFF baseline. This serial design preserves a joint-space policy interface while isolating transmission compensation from high-level motion learning.

The main contributions of this paper are as follows:

- **A transmission-aware hierarchical control architecture.** A simulation-trained reference-residual policy and an independently trained state-conditioned mapper are connected in series at deployment. The mapper converts desired joint positions and the measured plant state into motor-position commands, while the original fixed static calibration is retained as the Mapping-OFF baseline.
- **A globally anchored, multi-source, single-stage training pipeline.** Heterogeneous motions are retargeted to a common robot-space representation. Global-anchor rewards, motion- and segment-level adaptive sampling, and tendon-oriented domain randomization are integrated into one PPO stage that directly produces the deployable actor.
- **Randomized repeated physical validation.** Mapping OFF and ON are evaluated in randomized repeated trials on two nominally identical Droid X3 units. Within each robot–motion block, the frozen PPO checkpoint, reference motion, controller settings, safety bounds, and frozen mapper weights are held fixed; complete trials are used as the statistical units. Walk, squat, and dance provide multiple motion conditions, while the two separately assembled cable transmissions provide a cross-unit hardware check. The aggregate complete-trial action-completion rates are 68% for Mapping OFF and 79% for Mapping ON.

## 2. Related Work

### 2.1. Humanoid Motion Tracking

Reference-conditioned imitation and reinforcement learning provide a scalable route to humanoid whole-body control. Early studies reproduced dynamic human motions with dense pose rewards or adversarial motion priors [1,25]. More recent trackers include joint, link, root-velocity, and contact objectives and extend training from individual skills to large motion libraries or multiple embodiments [2,4,6,17,18].

These methods generally expose an idealized joint-space interface to the policy. Tendon-cable transmission weakens this assumption because elasticity, friction, backlash, hysteresis, calibration error, and coupling make the motor-to-joint response state dependent. On a full-size biped, transmission error also interacts with contact transitions and whole-body balance.

Our architecture retains a joint-space reference-residual policy but introduces a separately trained state-conditioned joint-to-motor mapper at the hardware interface. The encoder bias, response delay, equivalent stiffness/damping, and mapping residual are randomized during policy training; the physical mapper itself is trained offline and is absent from the simulation loop.

### 2.2. Multi-Source Motion Data and Global Consistency

Large motion libraries provide broad behavioral priors, but source-dependent skeletons, coordinate frames, sampling rates, and noise affect feasibility after retargeting. Recent work uses multi-source motion banks, video-derived interaction data, imperfect motion fragments, and diagnostic benchmarks to increase coverage and expose failure modes [13,15,21,22,23]. These findings motivate explicit robot-space normalization and data-quality filtering.

Long-horizon tracking further requires global spatial consistency. Studies of humanoid teleoperation and closed-loop tracking address retargeting, expert selection, localization feedback, and cumulative drift caused by gait error and contact disturbance [10,11,19,20,21].

We map all sources to a common Droid X3 representation containing joint states and key-link kinematics. A torso anchor constrains global position, orientation, and velocity, while anchor-relative link terms preserve whole-body geometry. The experiments evaluate stored reference sequences and physical execution; streaming teleoperation and external localization remain interface extensions rather than validated contributions.

### 2.3. Sim-to-Real Transfer via Domain Randomization

Large heterogeneous libraries exhibit long-tailed categories and nonuniform segment difficulty, while deployment introduces a separate simulation-to-reality gap. Prior work addresses these issues through adaptive sampling, closed-loop fine-tuning, data augmentation, and robust simulation design [13,18,22,23].

Domain randomization commonly perturbs mass, friction, inertia, and joint damping. For tendon-driven systems, the encoder bias, response delay, equivalent stiffness/damping, and mapping residual provide additional approximations to the calibration error, compliant lag, tension-dependent response, and coupling.

We combine motion-level failure and novelty sampling with segment-level hard-example sampling in a single PPO stage. Tendon-oriented perturbations are applied only to the simulated low-level response. The physical mapper is instead fitted from dedicated motor-excitation recordings and connected to the frozen policy at deployment.

### 2.4. Tendon-Cable Actuation

Compliant and indirect actuation has been studied in contact control, low-latency teleoperation, and safety-critical whole-body control [26,27,28], but explicit motor-to-joint mapping remains less developed for full-size tendon-cable-driven motion tracking. Configuration and transmission-state sensing provide a complementary perspective. Mao et al. [29], for example, study whole-body motion control for a six-bar tensegrity robot equipped with 24 flexible sensors; their framework also reconstructs robot shape and applies multimodal models to state recognition and fault diagnosis. Although related through flexible-cable state perception, configuration reconstruction, health monitoring, and whole-structure control, their substantially different robot morphology, sensing arrangement, and task formulation preclude treating the system as a directly comparable whole-body-control baseline for the present humanoid. Their sensing and diagnostic perspective motivates richer tendon-state sensing, online anomaly detection, and health monitoring as future extensions of the present framework.

Tendon-cable transmission combines remote actuation with compliant coupling, but the resulting elasticity, friction, backlash, hysteresis, tension drift, and multi-joint interaction violate independent-joint assumptions. Existing motion trackers typically address hardware mismatch through coarse randomization—for example, armature terms for reducer inertia in GMT [4] or velocity-dependent odometry noise in CLONE [11]—rather than an identified state-conditioned transmission map. This paper targets that interface on a full-size tendon-cable-driven biped.

## 3. Platform and Problem Formulation

### 3.1. The Droid X3 Platform

Droid X3 is a full-size humanoid engineering prototype developed by Shanghai DroidUp Co., Ltd. [24]. The experimental unit is approximately 1.68 m tall, has a measured mass of approximately 28 kg, and comprises a floating base and 28 actuated body joints distributed across the legs, waist, and upper limbs. Its actuated joints use chain–cable transmission.

The remote-transmission layout is intended to reduce distal-limb mass and inertia relative to joint-mounted actuation. Here, however, “lightweight” refers only to the measured total mass and this design intent; no segment-inertia comparison is reported. Similarly, compliance is a mechanical property rather than evidence of certified contact safety. The control-relevant consequence is that the same joint target can produce different responses under different transmission states, motion histories, and contact loads.

### 3.2. System Overview

The system comprises a robot-space motion library, a reference-residual policy, a state-conditioned joint-to-motor mapper, a fixed static-calibration baseline, and the physical low-level controller. At 50 Hz, the policy combines the current reference with proprioceptive history to produce a bounded residual joint target. During Mapping ON, the mapper converts this desired joint target and the measured plant state directly into a motor-position command. During Mapping OFF, the original fixed static calibration provides the motor-position command instead. The policy is trained in parallel MuJoCo simulations [30]; the mapper is fitted independently from physical motor-excitation trajectories.

This separation retains a common joint-space policy interface while localizing hardware-specific compensation to the mapper.

The closed-loop control pipeline operates at 50 Hz and proceeds in four stages per control step *t*. First, a reference command generator *G* queries the reference-motion sequence *M* at the current phase, conditioned on the robot state $x_{t}$, to produce a time-aligned motion command:

(1) $$g_{t}=G\left(M,t,x_{t}\right)=\left[\;q_{t}^{ref},\;{\dot{q}}_{t}^{ref},\;p_{t,B}^{ref},\;R_{t,B}^{ref},\;v_{t,B}^{ref},\;\omega_{t,B}^{ref}\;\right],$$

where $g_{t}$ aggregates the reference joint pose/velocity and the pose/velocity of the tracked body-link set *B*, serving as the high-level motion prior. Second, the reference-residual policy $\pi_{\theta }$ maps a length-*H* proprioceptive history $o_{t-H:t}^{p}$, the command $g_{t}$, and the previous action $a_{t-1}$ to a residual action:

(2) $$a_{t}∼\pi_{\theta }\;\left(a_{t}|o_{t-H:t}^{p},\;g_{t},\;a_{t-1}\right),\;a_{t}\in R^{n_{j}},$$

where $\pi_{\theta }$ is a Gaussian policy and $n_{j}$ is the number of actuated joints. Third, the residual is scaled by the per-joint range *S* and added to the reference joint target, then clipped to the soft joint limits to form the upper-layer desired joint target:

(3) $$q_{t}^{des}=\mathrm{clip}\;\left(q_{t}^{ref}+S⊙a_{t},\;q_{min}^{soft},\;q_{max}^{soft}\right),$$

where the reference prior $q_{t}^{ref}$ defines the nominal motion and *S* bounds the residual amplitude. Fourth, during physical Mapping ON, the independently trained mapper converts the desired joint target and current plant state into a motor-position command:

(4) $$x_{t}^{plant}=\left[q_{t-K:t}^{enc},\;p_{t-K:t}^{m},\;{\dot{p}}_{t-K:t}^{m},\;i_{t-K:t}^{m}\right],$$

(5) $$p_{t}^{m,map}=M_{\phi }\left(q_{t}^{des},x_{t}^{plant}\right),$$

(6) $$p_{t,r,\mathrm{ON}}^{m,cmd}=\mathrm{clip}\;\left(p_{t}^{m,map},p_{min}^{m},p_{max}^{m}\right).$$

Here, $q^{enc}$ denotes the measured joint position, while $p^{m}$, ${\dot{p}}^{m}$, and $i^{m}$ are the measured motor position, motor velocity, and motor current. The mapper output $p_{t}^{m,map}$ has motor-position units. Mapping OFF replaces this learned conversion with the robot’s fixed static calibration $C_{r}q_{t}^{des}+b_{r}$. The mapper is absent during nominal rigid-joint PPO training, where $q_{t}^{des}$ is applied directly as a simulated joint target; it is inserted only on the physical robot after PPO, the differentiable forward model, and the mapper have been trained and frozen. The measured hardware evidence comprises randomized repeated Mapping-OFF/ON trials on Robot 1 and Robot 2, as detailed in Section 5.3.

## 4. Method

**Figure 1:**
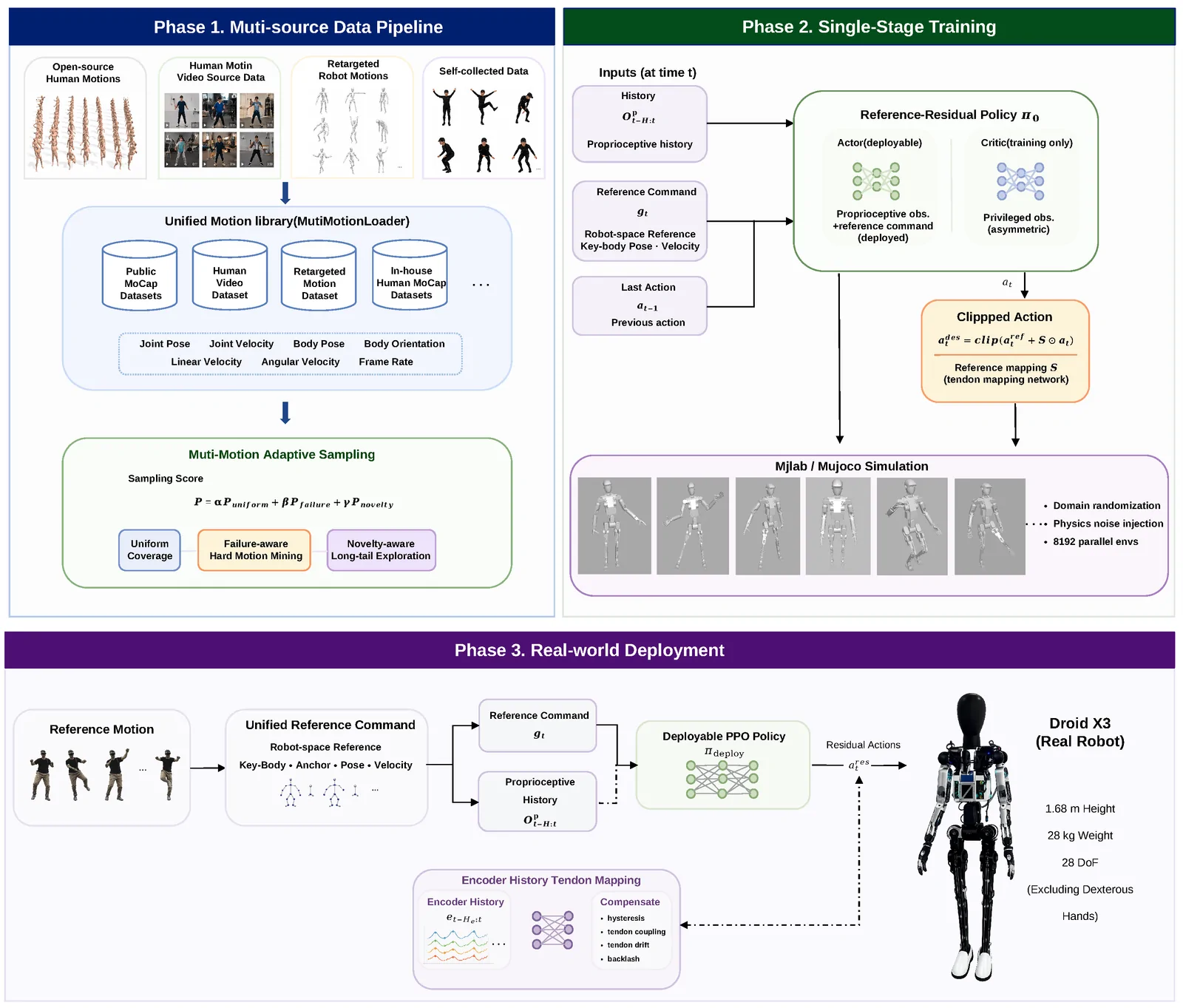
Overview of the proposed framework using the original three-phase layout. Phase 1 constructs the unified multi-source motion library and adaptive sampler. Phase 2 trains the reference-residual PPO policy in simulation; the reference mapping *S* denotes the fixed residual scaling, and the physical learned mapper is excluded from PPO. Before Phase 3 deployment, synchronized real-robot excitation data identify the differentiable forward model $F_{\psi }$, through which the state-conditioned mapper is trained offline and then frozen. During Mapping ON, the frozen mapper converts the complete policy-level desired-joint target and measured plant state into the complete motor-position command; Mapping OFF instead uses the fixed static calibration.

The high-level controller uses an asymmetric actor–critic: the actor receives deployable observations, whereas the critic additionally uses a privileged simulation state during PPO training and is discarded afterward. Physical excitation data first identify a differentiable motor–joint forward model; the tendon mapper is then trained separately through the frozen forward model and receives no PPO gradient. Figure 1 retains the original three-phase framework layout; the caption clarifies the separation between PPO training and offline mapper fitting.

### 4.1. Reference Motion Representation

Raw motions from open-source human MoCap, motion video, retargeted robot trajectories, and small-scale self-collected data are converted to the Droid X3 configuration by retargeting, forward kinematics, temporal resampling, and feasibility filtering. The public-source collection process included AMASS [31] and other open motion resources, but the final storage-group names do not retain a complete clip-level mapping to every upstream source. Exact reproduction of the processed library additionally requires clip identifiers, preprocessing parameters, and the historical split manifest; the recoverable archive statistics and remaining provenance limitations are reported in Section 5.1.

For a motion sequence of length *T*, the reference motion is represented as

(7) $$M={\left\{m_{t}\right\}}_{t=1}^{T},\;m_{t}=\left\{q_{t}^{ref},{\dot{q}}_{t}^{ref},p_{t,B}^{ref},R_{t,B}^{ref},v_{t,B}^{ref},\omega_{t,B}^{ref}\right\}.$$

Here, $q_{t}^{ref}$ and ${\dot{q}}_{t}^{ref}$ denote the robot joint position and velocity references; $p_{t,B}^{ref}$ and $R_{t,B}^{ref}$ denote the world-frame position and orientation of the key body-link set *B*; and $v_{t,B}^{ref}$ and $\omega_{t,B}^{ref}$ denote the corresponding linear and angular velocities.

The tracked links comprise the pelvis, bilateral thigh, shank, and foot links, the torso, and bilateral upper-arm, forearm, and wrist links. Combining joint and link targets constrains both local articulation and whole-body geometry, avoiding the under-constrained behavior of sparse end-point tracking.

To accommodate global-coordinate differences across data sources, training uses an anchor-aligned reference transform. Let the robot’s current anchor position and yaw be $\left(p_{t,A},\psi_{t,A}\right)$ and the reference anchor be $\left(p_{t,A}^{ref},\psi_{t,A}^{ref}\right)$. For any body link b∈B, the reference link pose is re-expressed relative to the current anchor through a yaw alignment:

(8) $$\mathrm{yaw}\mathrm{offset}:\;\Delta \psi_{t}=\psi_{t,A}-\psi_{t,A}^{ref},$$

(9) $$\mathrm{horizontal}\mathrm{offset}:\;\Delta p_{t,A}^{xy}={\left[\begin{array}{ccc} p_{t,A,x}-p_{t,A,x}^{ref} & p_{t,A,y}-p_{t,A,y}^{ref} & 0 \end{array}\right]}^{\;⊤},$$

(10) $$\mathrm{aligned}\mathrm{position}:\;{\hat{p}}_{t,b}^{ref}=p_{t,A}^{ref}+\Delta p_{t,A}^{xy}+R_{z}\left(\Delta \psi_{t}\right)\left(p_{t,b}^{ref}-p_{t,A}^{ref}\right),$$

(11) $$\mathrm{aligned}\mathrm{orientation}:\;{\hat{R}}_{t,b}^{ref}=R_{z}\left(\Delta \psi_{t}\right)\;R_{t,b}^{ref},$$

where $R_{z}$ denotes the yaw rotation about the world vertical axis. The translation term contains no vertical component; consequently, and the reference anchor is aligned to the current horizontal position, while its recorded height is preserved. This transform is used for anchor-relative link rewards. The global-anchor reward is evaluated against the unaligned reference anchor, so horizontal position and yaw errors are not removed from the global objective.

### 4.2. Observations, Policy, and Residual Action

We formulate whole-body motion tracking as a reference-conditioned Markov decision process:

(12) M=〈𝒮,𝒜,𝒫,ℛ,γ〉.

The policy observation consists of proprioceptive history, the reference-motion command, and the previous action:

(13) $$o_{t}=\left[o_{t-H:t}^{p},g_{t},a_{t-1}\right],\;a_{t}∼\pi_{\theta }\left(a_{t}|o_{t}\right).$$

The current implementation uses a 5-frame history. The actor observation contains projected gravity, IMU angular velocity, joint positions relative to the default pose, joint velocities, the previous action, reference joint position and velocity, reference base linear and angular velocity, the gravity direction under the reference pose, and the height of both feet relative to the base. Expanding the reference-command shorthand $g_{t}$, the deployment observation is

(14) $$o_{t}^{actor}={\left[q_{t}^{ref},\;{\dot{q}}_{t}^{ref},\;v_{A}^{ref},\;\omega_{A}^{ref},\;g_{A}^{ref},\;h_{feet}^{ref},\;g_{t}^{proj},\;\omega_{t}^{imu},\;q_{t}-q_{0},\;{\dot{q}}_{t},\;a_{t-1}\right]}_{t-H:t}.$$

The command includes velocity and foot-height cues in addition to pose, allowing the actor to infer motion phase and contact transitions. The history window provides short-term temporal context for sensing and execution lag.

The critic uses asymmetric observations, accessing more complete simulation-privileged information only during training. Beyond the actor observation, the critic can access noiseless anchor position error, anchor orientation error, body-link position/orientation, base linear velocity, reference base height, and key body-link states:

(15) $$o_{t}^{critic}=\left[o_{t}^{actor(noiseless)},e_{A}^{p},e_{A}^{R},p_{t,B},R_{t,B},v_{base},h_{A}^{ref},{\hat{p}}_{t,B}^{ref},{\hat{R}}_{t,B}^{ref}\right].$$

This design reduces the variance of value-function estimation during training while ensuring that the final deployed actor does not depend on simulation-privileged states.

We parameterize the action as “reference-sequence target plus network residual” rather than as raw joint torque or a fully free instantaneous command. For the tendon-cable-driven Droid X3, accurate torque inversion is difficult and susceptible to tension drift and backlash, while a fully free position command enlarges exploration and tends to produce high-frequency, non-smooth output.

The two learned components act at different interfaces. The policy residual refines a joint-space reference; downstream, the mapper converts the desired joint target and measured transmission state into the motor-position command. The fixed static calibration is retained as the Mapping-OFF baseline. The actor and mapper are trained separately and connected in series only at deployment.

The policy outputs a residual action $a_{t}$, and the nominal joint target follows Equation (3): the reference joint target $q_{t}^{ref}$ provides the main motion trend, while the scaled residual $S⊙a_{t}$ locally corrects current pose deviation, contact-state change, and retargeting error before clipping to the soft joint limits, forming the policy-level desired target $q_{t}^{des}$. In simulation, $q_{t}^{des}$ is directly executed by the joint-level PD controller. On the physical robot, the offline-trained mapper converts $q_{t}^{des}$ and the current plant state into a motor-position command before clipping, as defined in Equations (17)–(19). Thus, *S* limits the policy’s joint-space correction, whereas $M_{\phi }$ handles the subsequent joint-to-motor conversion under the tendon-transmission state.

The action scale *S* defines a conservative residual envelope. It is derived from actuator limits so that $S⊙a_{t}$ remains within the admissible deviation from $q_{t}^{ref}$ under the joint-level position servo. For each joint group *j*,

(16) $$S_{j}=\kappa_{j}\frac{\tau_{j}^{\mathrm{limit}}}{k_{j}},$$

where $\tau_{j}^{\mathrm{limit}}$ is the maximum continuous torque, $k_{j}$ the position-servo proportional gain, and $\kappa_{j}$ a conservative factor selected for the motor–tendon assembly. Under the quasi-static approximation $\tau \approx k_{j}\Delta q$, the constraint $k_{j}S_{j}\leq \kappa_{j}\tau_{j}^{\mathrm{limit}}$ bounds the torque induced by the residual offset.

Because the legs, waist, shoulders, forearms, and wrists differ in stiffness and torque rating, each joint group uses a separate $S_{j}$. This hardware-dependent scaling is more informative than a single global residual amplitude. The archived experiment record does not retain the group-wise numerical values of $S_{j}$, $\kappa_{j}$, $\tau_{j}^{\mathrm{limit}}$, and $k_{j}$; Equation (16) therefore documents the scale construction rather than a complete reproducible numerical configuration.

### 4.3. Tendon Mapping Network

For physical deployment, $M_{\phi }$ is a state-conditioned mapper that directly outputs a motor-position command. Its input comprises $q_{t}^{des}$ and a *K*-step history of measured joint position, motor position, motor velocity, and motor current:

(17) $$x_{t}^{plant}=\left[q_{t-K:t}^{enc},\;p_{t-K:t}^{m},\;{\dot{p}}_{t-K:t}^{m},\;i_{t-K:t}^{m}\right],$$

(18) $$p_{t}^{m,map}=M_{\phi }\left(q_{t}^{des},x_{t}^{plant}\right),$$

(19) $$p_{t,r,\mathrm{ON}}^{m,cmd}=\mathrm{clip}\;\left(p_{t}^{m,map},p_{min}^{m},p_{max}^{m}\right).$$

Joint history describes the execution error and motion direction, motor position and velocity describe the actuator-side state, and current provides a load-related proxy. The input $q_{t}^{des}$ is the complete desired-joint vector produced by the policy interface, including the reference target and policy residual defined in Equation (3). The mapper output $p_{t}^{m,map}$ is the complete motor-position command, not an additive correction to the static calibration, and is sent to the low-level motor controller after command clipping.

For the Mapping-OFF baseline, the mapper conversion is disabled and the original robot-specific static calibration is used:

(20) $$p_{t,r,\mathrm{OFF}}^{m,cmd}=\mathrm{clip}\;\left(C_{r}q_{t}^{des}+b_{r},\;p_{min}^{m},p_{max}^{m}\right),$$

where $C_{r}$ and $b_{r}$ are the fixed joint-to-motor calibration matrix and offset for unit *r*. In the retained Robot 1 comparison, OFF and ON therefore differ in the joint-to-motor conversion module while retaining the same frozen high-level PPO checkpoint and controller settings.

The mapper is trained offline from dedicated system-identification trajectories collected on the physical robot. For each motor in a coupled chain, multiple groups of bounded sinusoidal/cosinusoidal position commands are executed:

(21) $$p_{t,k,\ell }^{m,cmd}={\bar{p}}_{k,\ell }+A_{k,\ell }\sin\left(2\pi t/T_{k,\ell }\right)+B_{k,\ell }\cos\left(2\pi t/T_{k,\ell }\right),$$

where *k* denotes the excited motor and *ℓ* indexes the excitation group. Across groups, the center position ${\bar{p}}_{k,\ell }$, period $T_{k,\ell }$, and sine/cosine amplitudes $A_{k,\ell }$ and $B_{k,\ell }$ are varied within joint-specific safety limits. Varying the center and amplitudes changes the swept position interval, while varying the period changes the excitation speed; the resulting trajectory set therefore samples different positions, motion directions, and velocity levels within the safe operating range. During every real-robot trajectory, commanded and measured motor positions, motor velocity, motor current, and the encoder positions of all affected joints are acquired synchronously at the control rate. Exciting one motor while observing the complete coupled chain exposes both one-to-many and many-to-one transmission effects. Complete trajectories, rather than adjacent frames, form the intended partition unit so that phase, hysteresis, and motion direction remain intact. The archived record does not retain the historical training/validation/test counts, so no retrospective split is reported.

The synchronized trajectories form offline tuples $D={\left\{x_{t}^{plant},p_{t}^{m,cmd},q_{t+1}^{enc}\right\}}_{t=1}^{N}$. They are first used to identify a differentiable forward motor–joint model $F_{\psi }$:

(22) $${\hat{q}}_{t+1}^{enc}=F_{\psi }\left(x_{t}^{plant},p_{t}^{m,cmd}\right),$$

(23) $$L_{id}=\frac{1}{N}\sum_{t=1}^{N}{∥{\hat{q}}_{t+1}^{enc}-q_{t+1}^{enc}∥}_{2}^{2}.$$

After identification, $F_{\psi }$ is frozen. The mapper remains separate from PPO and is optimized through this differentiable forward model:

(24) $${\hat{p}}_{t}^{m,cmd}=\mathrm{clip}\;\left(M_{\phi }\left(q_{t+1}^{des},x_{t}^{plant}\right),\;p_{\min}^{m},p_{\max}^{m}\right),$$

(25) $${\hat{q}}_{t+1}^{map}=F_{\psi }\left(x_{t}^{plant},{\hat{p}}_{t}^{m,cmd}\right),$$

(26) $$L_{map}=\frac{1}{N}\sum_{t=1}^{N}{∥{\hat{q}}_{t+1}^{map}-q_{t+1}^{des}∥}_{2}^{2}.$$

Because ${\hat{q}}_{t+1}^{map}$ depends on $M_{\phi }$ through the frozen differentiable model $F_{\psi }$, the joint-space error is differentiable with respect to the mapper parameters. The forward model is used only during offline fitting. Deployment uses the frozen mapper for Mapping ON and the fixed static calibration for Mapping OFF. The current archive does not retain the network architecture, optimizer, split counts, or held-out identification errors for $F_{\psi }$ and $M_{\phi }$, so these quantities are not retrospectively specified.

This design preserves a unified joint-space policy while separating the learned state-conditioned joint-to-motor conversion from the static Mapping-OFF baseline. The mapper can address only states represented by its identification data; generalization to long-term wear, temperature drift, and unseen tension states is not established.

### 4.4. Reward Design and Termination

The reward combines global consistency, whole-body coordination, contact tracking, and command regularization. Tracking terms use the bounded kernel $K\left(e,\sigma \right)=\exp(-∥e{∥}_{2}^{2}/\sigma^{2})$, whereas penalty terms discourage abrupt action changes, joint-limit violations, and self-collision. These training constraints do not replace hardware-fault detection or emergency stopping.

The torso frame serves as the global anchor. Its position, orientation, and velocity constrain global motion, while averages over 14 tracked links constrain anchor-relative body geometry and velocity. Foot and base terms reinforce contact-relevant tracking. Table 1 lists the complete weighted objective.

An episode terminates at the end of the reference, at timeout, or when the anchor height, anchor orientation, or key-end-effector height exceeds a prescribed deviation. Failure terminations are fed to the adaptive sampler to increase the probability of the associated motion and temporal segment.

### 4.5. Adaptive Sampling and Domain Randomization

Reference libraries are typically imbalanced in category frequency, clip length, difficulty, and source quality. Uniformly sampling such a library can overexpose easy locomotion, whereas emphasizing the hardest segments too early can destabilize PPO updates.

The loader packages joint and link kinematics into contiguous tensors and supports CPU/GPU preloading, cross-GPU sharding, and optional motion-group partitioning. The archived runs use cross-GPU sharding but do not specify motion groups or group-level ratios; group balancing is therefore an available interface rather than a reported active factor.

Motion-level sampling mixes uniform coverage, failure-based difficulty, and novelty. For motion *i*, the capped failure score $D_{i}$, novelty score $N_{i}$, and uniform score $U_{i}$ are

(27) $$f_{i}=\frac{n_{i}^{fail}}{\max(n_{i}^{sample},1)},\;D_{i}=\mathrm{Norm}\left(\min\left(f_{i},\beta_{cap}\right)\right),$$

(28) $$N_{i}=\mathrm{Norm}\left(\frac{1}{\sqrt{n_{i}^{assigned}+1}}\right),\;U_{i}=\frac{1}{N}.$$

A warmup-and-ramp factor ρ(t) schedules the nonuniform weights:

(29) $$w_{f}\left(t\right)=\rho \left(t\right)w_{f}^{target},\;w_{n}\left(t\right)=\rho \left(t\right)w_{n}^{target},\;w_{u}\left(t\right)=1-w_{f}\left(t\right)-w_{n}\left(t\right).$$

The final motion-level sampling probability is

(30) $$p_{i}=\mathrm{Norm}\left(w_{u}\left(t\right)U_{i}+w_{f}\left(t\right)D_{i}+w_{n}\left(t\right)N_{i}\right).$$

When the library is partitioned into motion groups by source or category, group-level probabilities can be applied:

(31) P(i)=P(group(i))P(i∣group(i)).

Within each motion, temporal bins maintain failure and tracking-error statistics. An exponential moving average and an optional smoothing kernel define the segment probabilities:

(32) $$F_{i,b}\leftarrow \alpha C_{i,b}+\left(1-\alpha \right)F_{i,b},$$

(33) $${\tilde{F}}_{i,b}=\mathrm{SmoothKernel}\left(F_{i,b};\lambda ,k\right),\;p_{i,b}=\mathrm{Norm}\left({\tilde{F}}_{i,b}+\epsilon /B_{i}\right).$$

Here, $F_{i,b}$ is the difficulty estimate for bin *b* of motion *i*, $C_{i,b}$ is its current failure or error signal, and $B_{i}$ is the number of bins. The archived configuration uses α=0.001, λ=0.8, a uniform mixture of 0.1, target failure and novelty weights of 0.5 and 0.3, $\beta_{cap}=2.0$, a 100 s warmup, and a 300 s cosine ramp. Its kernel size is k=1, so SmoothKernel reduces to the identity and does not spread probability to neighboring bins in the reported runs.

The resulting hierarchy separates library coverage from within-motion difficulty: motion probabilities preserve nonzero coverage, segment probabilities focus on difficult intervals, and the schedule limits early overconcentration. Section 5.5 reports the available archived training diagnostics; because the retained runs use different motion archives, they are not treated as a controlled isolation of individual sampling terms.

**Domain randomization.** The randomized parameter vector ξ∼P(ξ) perturbs the reference, robot body, contact, observations, and equivalent tendon execution, giving $x_{t+1}=f_{\xi }\left(x_{t},u_{t},c_{t}\right)$. Tendon execution is approximated through response delay, stiffness/damping scaling, and mapping residual; the physical learned mapper is not evaluated in simulation. Table 2 lists the recorded ranges.

At reset, reference pose and joint targets are perturbed and clipped to the soft limits. External velocity disturbances, torso center-of-mass offsets, encoder bias, and foot-friction changes broaden the simulated operating range. Tendon-execution perturbations expose the policy to low-level response mismatch without asserting direct coverage of measured physical tension states.

### 4.6. Single-Stage Training and Deployment

We train a deployable actor directly with single-stage proximal policy optimization (PPO) [32]. PPO is an on-policy actor–critic algorithm that updates the policy using a clipped probability-ratio objective, limiting the size of each update relative to the data-collecting policy. “Single stage” refers specifically to optimization of the high-level reference-residual actor and critic in the simulation without a subsequent teacher-to-student distillation stage. The tendon mapping network is not part of the PPO computation graph: it is trained independently and offline from real-robot trajectory recordings after policy training, and is subsequently connected in series between the frozen policy output and the robot-side low-level controller during deployment.

We optimize the policy with PPO using a clipped surrogate objective, where the advantage ${\hat{A}}_{t}$ is computed by generalized advantage estimation (GAE) [33]. With actor parameters θ and critic parameters η, the combined objective is maximized:

(34) $$\begin{array}{cc} J\left(\theta ,\eta \right)=E_{t}[\; & \min\left(\rho_{t}\left(\theta \right){\hat{A}}_{t},\;\mathrm{clip}\left(\rho_{t}\left(\theta \right),1-\epsilon ,1+\epsilon \right){\hat{A}}_{t}\right)\\ & \;-c_{v}{\left(V_{\eta }\left(s_{t}\right)-{\hat{R}}_{t}\right)}^{2}+c_{h}H\left[\pi_{\theta }\left(\cdot |o_{t}\right)\right]], \end{array}$$

where the probability ratio, GAE advantage, and temporal-difference residual are

(35) $$\begin{array}{cc} \rho_{t}\left(\theta \right) & =\frac{\pi_{\theta }\left(a_{t}|o_{t}\right)}{\pi_{\theta_{\mathrm{old}}}\left(a_{t}|o_{t}\right)},\;{\hat{A}}_{t}=\sum_{l=0}^{T-t-1}{\left(\gamma \lambda \right)}^{l}\;\delta_{t+l},\\ \delta_{t} & =r_{t}+\gamma V_{\eta }\left(s_{t+1}\right)-V_{\eta }\left(s_{t}\right). \end{array}$$

where $\rho_{t}\left(\theta \right)$ is the probability ratio between the current and previous policies, ϵ the clipping range, ${\hat{A}}_{t}$ the GAE advantage with TD residual $\delta_{t}$, and γ and λ the discount and GAE factors; H[·] is the policy-entropy bonus, and $c_{v}$ and $c_{h}$ weight the value loss and entropy regularization. The critic is trained on asymmetric privileged observations to reduce value-estimation variance, while the actor consumes only proprioceptive history and reference commands available at deployment.

The actor and critic are both multilayer perceptrons with hidden sizes of 1024, 1024, 512, and 256, ELU activations, and running observation normalization; the action distribution is Gaussian with an initial standard deviation of 1.0. Training runs 8192 parallel environments in MuJoCo with a rollout length of 24 steps and up to $3\times 10^{5}$ iterations. The physics step is 0.005 s with a control decimation of 4, yielding a 50 Hz policy command-update rate. The serial actor–mapper path has been run in the physical closed loop on the RK3588, while separate offline inference was checked on the RTX 4090. Per-module mean/tail latency, jitter, resident/peak memory, and deadline-miss rate are not numerically reported. The key hyperparameters are summarized in Table 3.

Unlike frameworks that first train a privileged teacher and then distill a deployable student [4,5], our implementation directly optimizes the deployable actor. The reference-sequence target supplies a motion prior, hierarchical adaptive sampling concentrates updates on hard motions and failure segments, and domain randomization introduces robustness perturbations during the same actor–critic optimization stage. This is an architectural and workflow distinction only: without a teacher–student implementation matched for observations, architecture, training samples, and tuning budget, the present paper does not claim a quantitative performance advantage over distillation.

**Deployment interface and evaluated scope.** The reported experiments replay complete robot-space reference sequences spanning locomotion, bending, dance, and coordinated upper-limb motion. Tracking failures update the data-quality and adaptive-sampling statistics. A future streaming or task-level front end could use the same command representation, but this interface compatibility is not an end-to-end validation.

No streaming teleoperation, input-delay/jitter, packet-loss, missing-pose, operator-workload, or usability experiment is reported.

External localization and world-frame drift correction are also outside the evaluated system; the anchor reward alone is not evidence of localization-based drift rejection.

## 5. Experiments

This section reports the qualitative multi-category tracking evidence, physical multi-robot mapper records, the implemented policy design, and archived adaptive-sampling diagnostics. Numerical entries that could not be traced to an archived experimental record have been removed rather than reconstructed.

### 5.1. Experimental Setup

**Platform and simulation.** Policies are trained in parallel MuJoCo/MjLab environments with a 0.005 s physics step and a control decimation of four, yielding a 50 Hz command rate. Table 3 lists the PPO configuration. Physical trials use the Droid X3 platform described in Section 3.1; all reported physical records were collected indoors on a level laboratory floor.

**Dataset audit and split provenance.** Table 4 reports the recoverable composition of the processed archive: 94,359 source sequences and 39,482,115 non-overlapping frames at 50 Hz (219.345 h). Sequences are packaged into 127,091 overlapping windows of at most 512 frames using a regular stride of 448 frames and an endpoint-aligned final window. The windows contain 46,135,401 frames in total, but this repeated-frame count is not interpreted as independent data duration. Public subsets can be regenerated from their cited sources, whereas Pico-record and small self-collected subsets require the corresponding local recordings. The robot-space library is produced by retargeting, temporal resampling, conditional feasibility screening, and windowing. The screening conditions comprise a prescribed robot-height envelope, finite-difference joint-acceleration checks, and robot joint-limit checks. The exact numerical thresholds, historical source-level split manifest, clip identifiers, mapper trajectory-level split manifest, and preprocessing-script revision associated with the reported frozen checkpoint were not retained. The processing stages can therefore be reimplemented for available source data, but neither the complete historical library nor the checkpoint’s training assignment can be reconstructed exactly from the public sources alone. Consequently, the existing checkpoint is not presented as a same-source-unseen or cross-source benchmark.

The archived evaluation categories include walking/turning, deep squat/bend, high-leg/kick, dance/martial arts, large upper-limb motion, coordinated arm–leg motion, and long sequences. Simulation evaluation of the trained frozen checkpoint over 12,674 tested reference motions achieved an aggregate action-completion rate above 90%. This is a descriptive checkpoint-level result over the tested collection. Because the historical source-level assignment manifest was not retained, it is not interpreted as a source-disjoint or cross-source generalization statistic.

**Evaluation metrics.** The tracking metrics used are mean deviations from the reference: torso-anchor position and yaw errors ($E_{anchor}^{pos}$, $E_{anchor}^{yaw}$), anchor-relative link-position error $E_{body}^{pos}$, link-velocity error $E_{vel}$, and joint error $E_{joint}$. The action rate is $E_{action}=T^{-1}\sum_{t}{∥a_{t}-a_{t-1}∥}_{2}^{2}$. A rollout succeeds if it completes the reference without a failure termination. Physical trials additionally report per-joint position MAE and torso-orientation error, as defined in Section 5.3.

**Experimental protocol.** Category-level tracking uses complete references from the archived evaluator. Because the original per-category and per-ablation rollout manifests were not retained, no reconstructed numerical category or policy-ablation results are reported. The adaptive-sampling subsection compares three archived training records that use different motion archives and therefore reports training diagnostics rather than a controlled one-factor ablation. Independent-seed metadata for the archived simulation runs are unavailable, so no simulation-side inferential statistics are reported. The verified aggregate action-completion rate above 90% over 12,674 tested reference motions is retained as a descriptive result of frozen-checkpoint evaluation, without a cross-source or significance claim. The physical protocol uses the same frozen PPO checkpoint, reference motions, controller settings, safety bounds, and frozen mapper weights on two nominally identical Droid X3 units. Within each robot–motion block, complete Mapping-OFF/ON trials are repeated and their order is randomized; a complete trial, rather than an individual 50 Hz frame or a displayed temporal segment, is the statistical unit. OFF uses the fixed conversion in Equation (20), whereas ON uses the learned conversion in Equation (19). The figures below show randomly selected temporal segments for time-resolved visualization and are not used as independent replicates.

**Closed-loop timing scope.** The actor–mapper command path is configured to execute serially on the on-robot RK3588 at a 50 Hz control rate (20 ms period). The retained reference, command, and feedback logs are also sampled at 50 Hz and therefore cannot resolve within-cycle inference, communication latency, or jitter. The configured control rate is reported as an implementation setting, not as a worst-case deadline guarantee; sub-cycle latency, memory, and deadline-miss distributions were not measured.

### 5.2. Complex Whole-Body Motion Tracking

**Figure 2:**
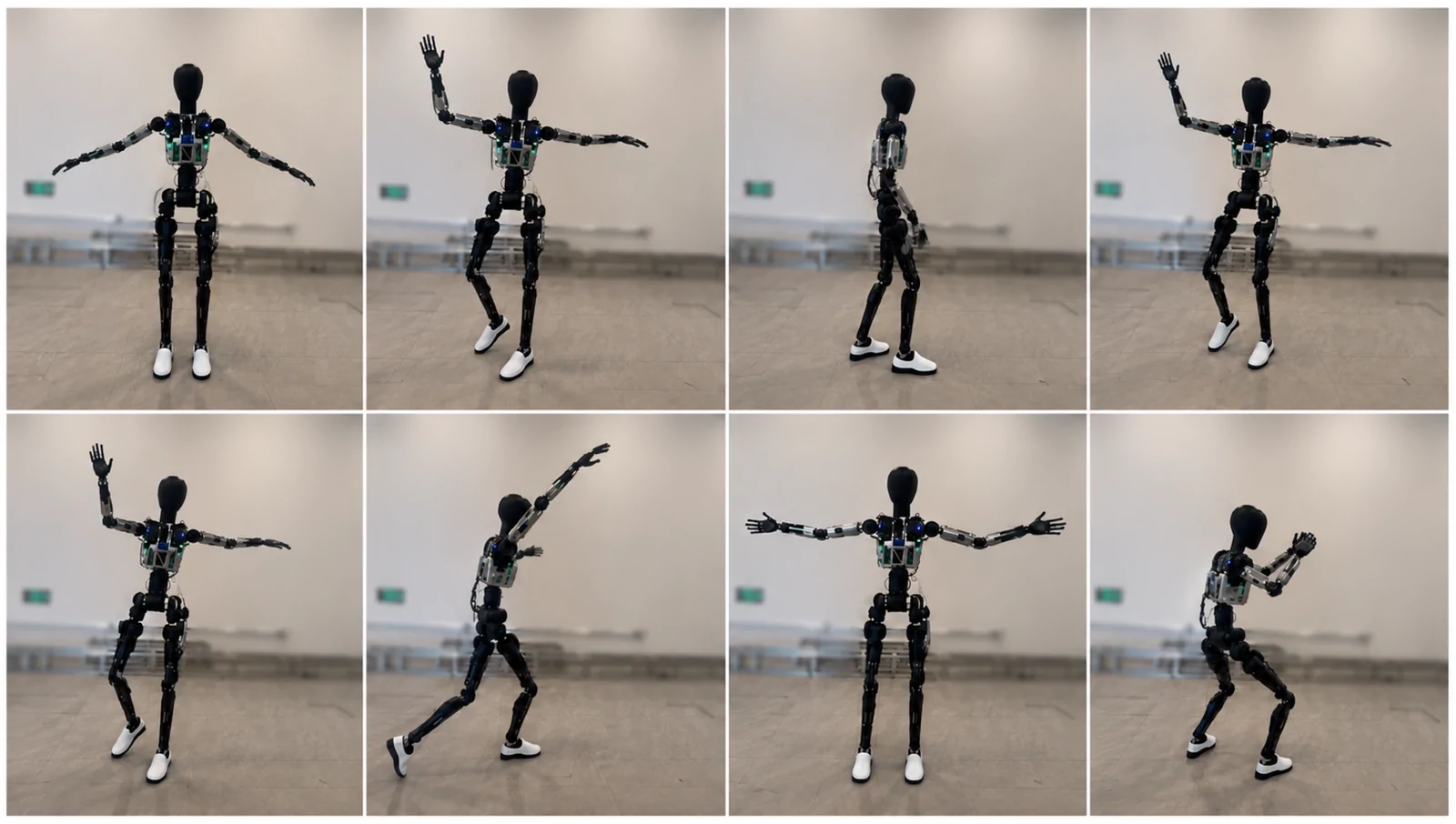
Representative physical poses under one reference-residual controller, illustrating large upper-limb excursions and coordinated arm–leg postures. These are qualitative frames rather than standardized gesture classes or independent samples; quantitative metrics are computed over complete reference sequences.

The archived evaluator contains complete references spanning walking and turning, deep squat and bending, high-leg and kicking motions, dance and martial-arts motions, large upper-limb excursions, coordinated arm–leg motion, and long sequences. The original per-category rollout manifest is not available. In simulation checkpoint evaluation over 12,674 tested reference motions, the trained frozen checkpoint achieves an aggregate action-completion rate above 90%, where completion requires reaching the end of the reference without a failure termination. We report this aggregate descriptive result and the evaluated motion scope, but do not infer source-wise generalization or statistical significance. We therefore retain qualitative physical poses, but do not retain the earlier difficulty-scaled numerical category table or infer category ordering from reconstructed values. Figure 2 provides representative physical poses.

The robot also completed a sequential batch of heterogeneous references without losing tracking before the final motion. This is an operational-continuity observation for that session, not a standardized endurance result; uninterrupted duration, interventions, thermal evolution, and pre/post-transmission error were not recorded.

### 5.3. Tendon Mapping Validation and Transmission Characterization

Randomized repeated Mapping-OFF/ON trials are conducted on Robot 1 and Robot 2 using multiple types of physical reference trajectories. The two units have the same dimensions, link geometry, 28-DoF configuration, and tendon-cable layout, but their cable transmissions are assembled independently. Within each robot–motion block, the frozen high-level PPO checkpoint, reference motion, controller settings, safety bounds, and frozen mapper weights are held fixed, and the OFF/ON order is randomized. OFF uses the unit-specific fixed conversion $C_{r}q_{t}^{des}+b_{r}$, whereas ON feeds the complete policy-level desired-joint vector $q_{t}^{des}$ and measured plant state to $M_{\phi }$, which directly outputs the complete motor-position command. The mapper is not retrained or fine-tuned for Robot 2. The statistical analysis includes the complete repeated trials across the tested reference types. The plotted walk, squat, and dance trajectories are randomly selected temporal fragments from those physical trials; unequal frame counts indicate different visualization boundaries rather than different checkpoints or experimental units.

Representative physical executions and Mapping-OFF/ON comparisons are provided in Video S1.

**Figure 3:**
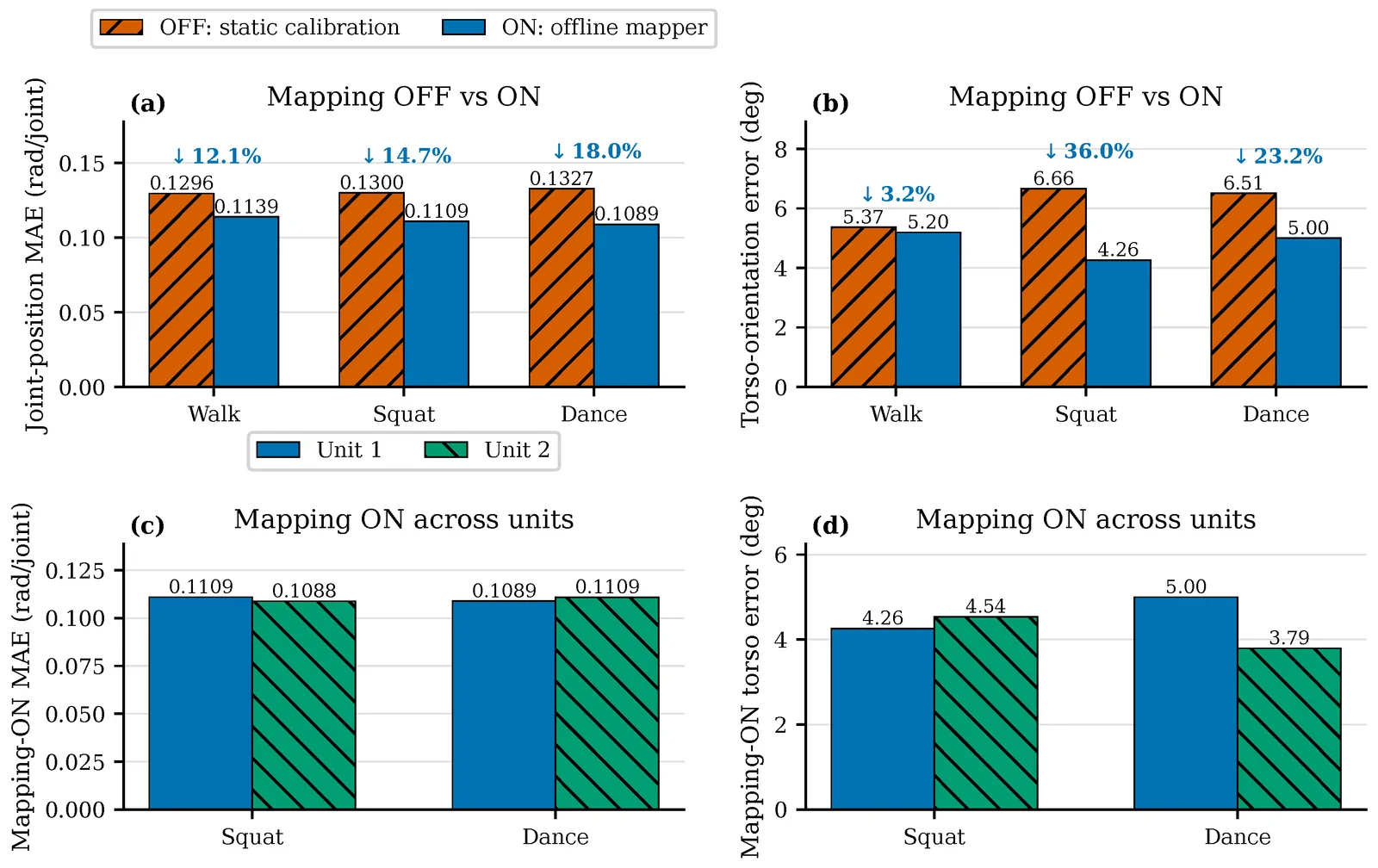
Physical tracking-error summary for randomly selected trajectory fragments from the repeated trial sets. Panels (**a**,**b**) compare Robot 1 examples using the static joint-to-motor conversion (OFF) and the learned mapper conversion (ON). Under Mapping ON, the frozen mapper receives the complete policy-level desired-joint vector and measured plant state and directly outputs the complete motor-position command. Panels (**c**,**d**) compare measured physical Mapping-ON examples from Robot 1 and Robot 2 using identical frozen mapper weights. Complete randomized trials, rather than these displayed fragments, are the statistical units. Downward arrows above the bars indicate the relative reduction in the displayed tracking error from Mapping OFF to Mapping ON.

Figure 3 summarizes the physical tracking-error comparisons and the cross-unit Mapping-ON records.

At the complete-trial level, the aggregate action-completion rate is 68% for Mapping OFF and 79% for Mapping ON, corresponding to an increase of 11 percentage points. Completion requires reaching the end of the full physical reference trajectory without a failure termination. These condition-specific physical results are distinct from the above-90% checkpoint-level completion result over 12,674 tested reference motions. The completion rates are reported descriptively; no statistical-significance claim is made.

**Multi-motion and multi-robot comparison.** The displayed temporal-segment records are summarized using per-joint trajectory MAE:

(36) $$E_{\mathrm{joint}}^{\mathrm{MAE}}=\frac{1}{TJ}\sum_{t=1}^{T}\sum_{j=1}^{J}\left|q_{t,j}^{\mathrm{ref}}-q_{t,j}^{\mathrm{enc}}\right|,$$

where J=28. The torso-orientation error is the mean geodesic angle between reference and measured orientations. Each displayed metric is computed over all frames of the selected trajectory fragment. The repeated-trial evaluation uses each complete trial as one independent unit to check consistency across reference types and robot units; the 50 Hz frames and displayed fragments are not treated as independent replicates or as a basis for a significance claim.

**Figure 4:**
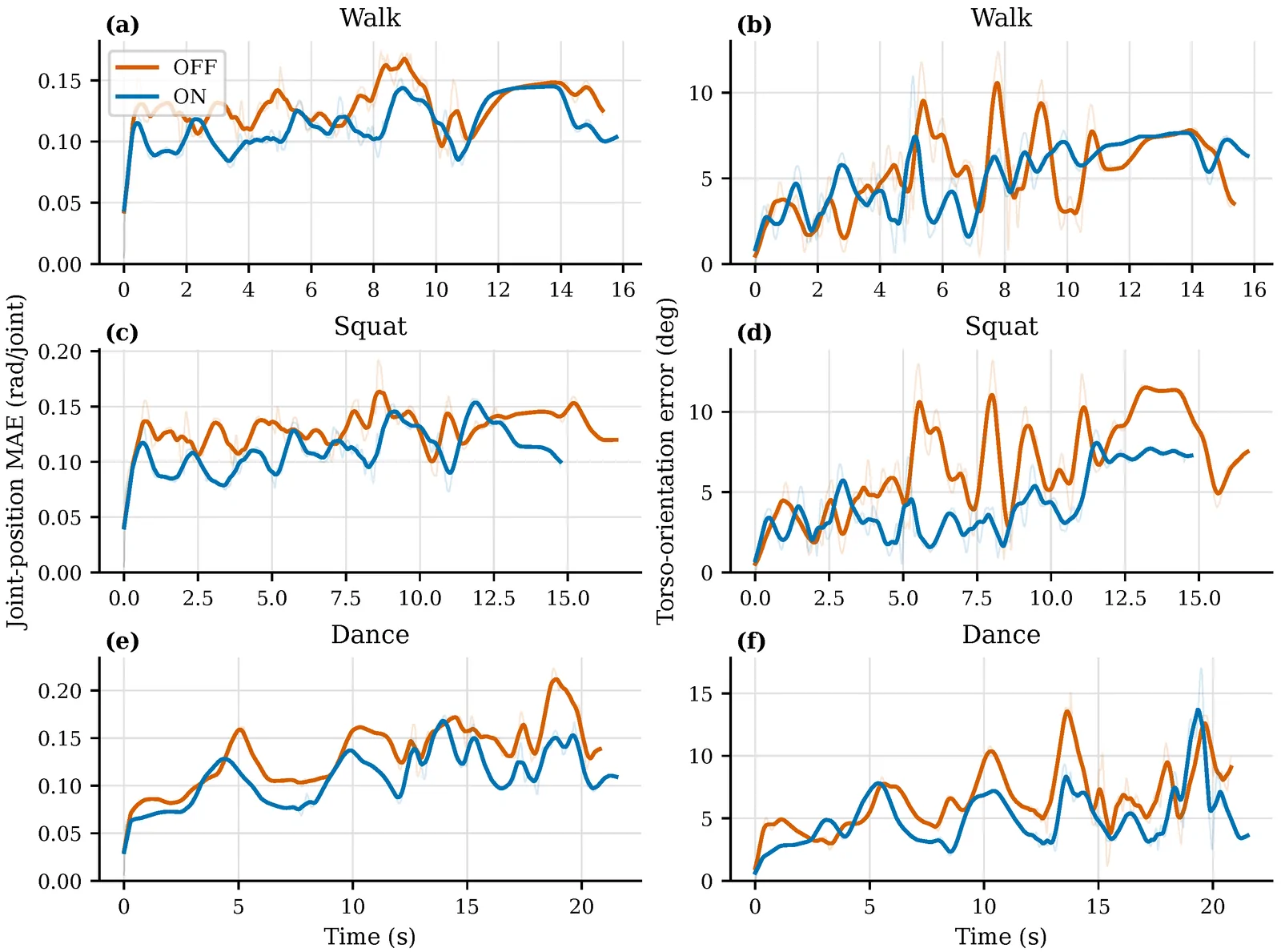
Time-resolved real-robot tracking errors for randomly selected walk, squat, and dance fragments with Mapping OFF and ON: (**a**) walk joint-position MAE; (**b**) walk torso-orientation error; (**c**) squat joint-position MAE; (**d**) squat torso-orientation error; (**e**) dance joint-position MAE; and (**f**) dance torso-orientation error. Thin curves show per-frame errors; thick curves show 25-frame (0.5 s) moving averages. Statistical pairing is performed at the complete-trial level, not frame by frame.

Table 5 lists the corresponding segment summaries, and Figure 4 resolves the Robot 1 OFF/ON records in time to show phase-dependent error trends for all three motions.

In the displayed Robot 1 fragments, the ON trajectory is lower than the corresponding OFF trajectory by 12.1%, 14.7%, and 18.0% in joint-position MAE for walk, squat, and dance; the corresponding illustrative torso-orientation differences are 3.2%, 36.0%, and 23.2%. Their unweighted fragment-level means are 0.1308 and 0.1113 rad/joint and $6.179^{\circ }$ and $4.820^{\circ }$, respectively. These numbers visualize representative trajectories and are not substituted for the randomized complete-trial statistics. The displayed physical Robot 2 Mapping-ON fragments yield 0.1088 and 0.1109 rad/joint for squat and dance, respectively; the corresponding torso-orientation errors are $4.538^{\circ }$ and $3.795^{\circ }$.

### 5.4. Policy-Side Design Scope

The implemented policy combines the robot-space reference, a learned residual, and action-rate regularization. The earlier manuscript included numerical rows for direct-target, reference-only, and residual-only variants that were not linked to independent archived runs. Those reconstructed values and the associated table have been removed. Consequently, the present paper describes the implemented policy structure but does not claim a controlled factorial ablation of reference conditioning, residual learning, smoothing, and mapper effects. The physical mapper is absent from PPO training and is evaluated separately by the randomized physical Mapping-OFF/ON protocol in Section 5.3.

### 5.5. Single-Stage Training Diagnostics and Adaptive Sampling

The retained records provide three single-stage PPO training runs on different motion archives. Their adaptive-sampling parameters are the same, so the runs characterize training behavior under different archive compositions rather than isolate uniform, motion-level, and segment-level sampling factors. The earlier interpolated motion-level-only row has been removed because it was not an independent run. Table 6 summarizes the archived training-run diagnostics.

**Figure 5:**
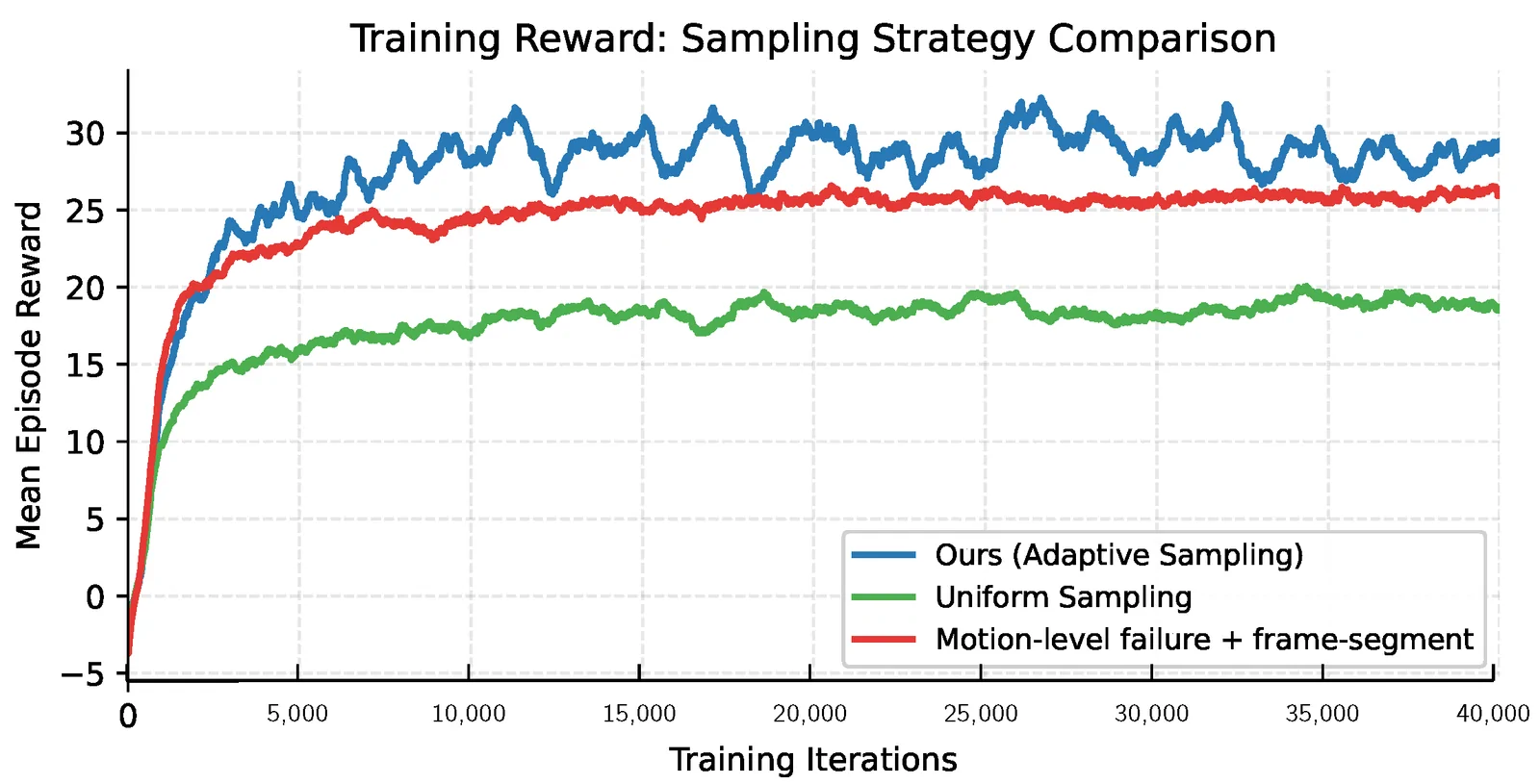
EMA-smoothed training reward for the three archived runs on different motion archives. Archive C has the highest retained final reward and Archive B reaches reward 20 first. Legacy labels embedded in the plot identify the original records but do not establish a controlled sampling ablation.

**Figure 6:**
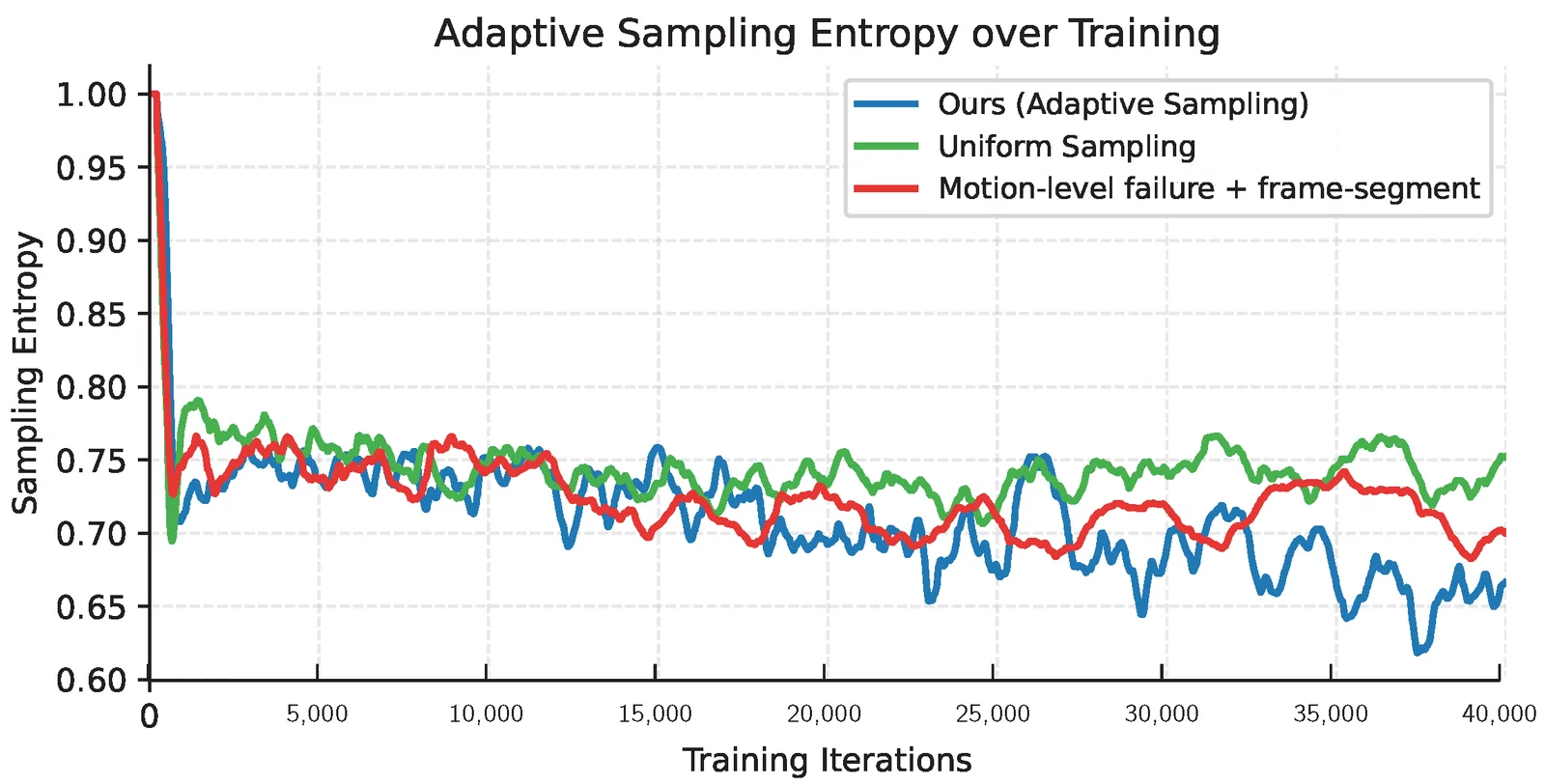
Sampling entropy for the three archived runs. The curves document nonzero sampling diversity under their respective motion archives; differences cannot be assigned to a single sampling factor because the archive composition is not controlled. Legacy labels embedded in the plot identify the original records only.

Archive A reaches reward 20 at 10,551 iterations, Archive B at 1412 iterations, and Archive C at 1604 iterations. Archive C has the highest retained mean reward and the lowest final sampling entropy among these runs. Because the motion archives differ, the numerical gaps cannot be attributed solely to a sampling component. Figure 5 and Figure 6 show the three archived training curves; their legacy legend names are run identifiers rather than validated one-factor ablation labels.

## 6. Conclusions and Discussion

We presented a hierarchical whole-body tracking framework for the 28-DoF Droid X3. A reference-residual actor is trained in simulation from a unified, globally anchored motion representation, while a separately trained mapper converts desired joint targets and measured plant state into motor-position commands. The static calibration provides the Mapping-OFF baseline. This separation preserves a common policy representation across motion sources and confines transmission compensation to the deployment layer.

In simulation evaluation over 12,674 tested reference motions, the trained frozen checkpoint achieves an aggregate action-completion rate above 90%. This checkpoint-level result is descriptive because the retained archive does not support a retrospective source-disjoint or cross-source breakdown.

Among the three retained training runs, Archive C has the highest final reward-derived score and the lowest sampling entropy; the runs use different motion archives and therefore do not constitute a controlled sampler ablation. The physical study uses randomized repeated Mapping-OFF/ON trials on two nominally identical Droid X3 units while holding the PPO checkpoint, mapper weights, reference motions, and controller settings fixed. The complete-trial action-completion rate is 68% for Mapping OFF and 79% for Mapping ON. The time-series figure and per-trajectory table report randomly selected physical segments for visualization; trial-level statistics use complete repeated trials as the independent units.

The modular architecture allows the motion library, sampling process, and hardware mapper to be revised without changing the high-level joint-space interface.

### Scope and Limitations

**Dataset provenance and reproducibility.** The archive-level source, frame, duration, and window counts are recoverable, but the historical train/validation/test manifest, complete clip-to-upstream-source mapping, numerical feasibility thresholds, preprocessing-script revision, and mapper trajectory-level split manifest are unavailable. The documented processing stages support method-level reimplementation on available source data, but do not support a byte-identical reconstruction of the complete historical library or frozen checkpoint. A future release should include versioned source and split manifests, fixed thresholds, preprocessing code, and complete-trajectory mapper partitions.

**Hardware and mapper coverage.** Randomized repeated Mapping-OFF/ON trials were conducted on Robot 1 and Robot 2 using the same frozen PPO checkpoint and identical frozen mapper weights. Conditions were compared within robot and motion so that unit-specific cable transmission did not replace the within-unit mapper toggle. Complete trials are the independent statistical units; the displayed temporal segments are illustrative only. The two units have the same dimensions, link geometry, 28-DoF configuration, and tendon-cable layout, while their separately assembled cable transmissions provide unit-specific hardware conditions. Across the evaluated complete physical trials, the aggregate action-completion rates are 68% for Mapping OFF and 79% for Mapping ON. Pretension, temperature, wear, payload, and motion-speed strata were not directly controlled, so the cross-unit result is not interpreted as a controlled test of any one of these physical factors.

**Environment and duration.** The evaluation is limited to indoor level-floor reference tracking. Outdoor navigation, uneven or deformable terrain, long-distance navigation, complex obstacle negotiation, load carrying, and contact-rich manipulation are not evaluated. Completion of a heterogeneous motion batch provides only a session-level continuity check; total uninterrupted duration, intervention count, thermal evolution, long-duration cable wear, actuator degradation, calibration drift, recalibration intervals, and pre/post-transmission error were not quantified. Scheduled re-identification, richer transmission-state sensing, online anomaly detection, and health monitoring [29] are relevant future directions.

**Teleoperation and user interaction.** The robot-space interface can accept future streaming commands, but no end-to-end teleoperation, input-delay/jitter/dropout benchmark, missing-pose recovery test, operator study, NASA-TLX/SUS assessment, or standardized human–robot-interaction evaluation is reported. Accordingly, real-time teleoperation and operator usability are not claimed as validated contributions.

**Computation.** The serial actor–mapper path is configured at 50 Hz on the RK3588, but the retained 50 Hz traces cannot resolve within-cycle timing. P50/P95/P99 latency, jitter, memory, deadline misses, and scaling are unreported; the configured update rate is not presented as a real-time deadline benchmark.

**Safety and failure handling.** Action clipping and training penalties do not constitute a field-safety system. This study does not evaluate emergency-stop latency, watchdog and communication-timeout behavior, current/temperature protection, cable-break detection, fall protection, recovery control, or human-contact safety. These hardware-level mechanisms, thresholds, and response times require separate validation before outdoor, long-horizon, or close-proximity deployment.

## Figures and Tables

**Table 1:** Tracking rewards and regularization penalties. *A* is the torso anchor, *B* is the set of 14 tracked links, $d_{R}$ is geodesic rotation error, and superscript “rel” denotes anchor-relative coordinates.

Term	Definition	Weight
Global-anchor position	$\exp\;\left(-∥p_{A}-p_{A}^{ref}{∥}_{2}^{2}/0.3^{2}\right)$	0.5
Global-anchor orientation	$\exp\;\left(-d_{R}{\left(R_{A},R_{A}^{ref}\right)}^{2}/0.4^{2}\right)$	0.5
Global-anchor linear velocity	$\exp\;\left(-∥v_{A}-v_{A}^{ref}{∥}_{2}^{2}/1.0^{2}\right)$	1.0
Key-body position	$\frac{1}{\left|B\right|}\sum_{b\in B}\exp\;\left(-∥p_{b}^{rel}-{\hat{p}}_{b}^{ref,rel}{∥}_{2}^{2}/0.3^{2}\right)$	1.0
Key-body orientation	$\frac{1}{\left|B\right|}\sum_{b\in B}\exp\;\left(-d_{R}{\left(R_{b}^{rel},{\hat{R}}_{b}^{ref,rel}\right)}^{2}/0.4^{2}\right)$	1.0
Key-body linear velocity	$\frac{1}{\left|B\right|}\sum_{b\in B}\exp\;\left(-∥v_{b}-v_{b}^{ref}{∥}_{2}^{2}/1.0^{2}\right)$	1.0
Key-body angular velocity	$\frac{1}{\left|B\right|}\sum_{b\in B}\exp\;\left(-∥\omega_{b}-\omega_{b}^{ref}{∥}_{2}^{2}/\pi^{2}\right)$	1.0
Feet position	$\exp\;\left(-∥p_{F}-p_{F}^{ref}{∥}_{2}^{2}/0.5^{2}\right)$	0.8
Base orientation	$\exp\;\left(-d_{R}{\left(R_{\mathrm{base}},R_{\mathrm{base}}^{ref}\right)}^{2}/0.5^{2}\right)$	0.5
Base linear velocity	$\exp\;\left(-∥v_{\mathrm{base}}-v_{\mathrm{base}}^{ref}{∥}_{2}^{2}/1.0^{2}\right)$	0.5
Base angular velocity	$\exp\;\left(-∥\omega_{\mathrm{base}}-\omega_{\mathrm{base}}^{ref}{∥}_{2}^{2}/\pi^{2}\right)$	0.5
Action smoothness	$∥a_{t}-a_{t-1}{∥}_{2}^{2}$	−0.3
Joint limit	$n_{\mathrm{limit}}\left(q_{t}\right)$	−10
Self-collision	$n_{\mathrm{collision}}$	−10

**Table 2:** Domain-randomization configuration. Episode-level variables are sampled at reset and observation noise is sampled per control step. Multiplicative ranges are marked by ×.

Layer	Parameter	Range/Unit
Reference input	Root position (x,y,z)	±0.05/±0.05/±0.01 m
	Root orientation (r/p/y)	±0.1/±0.1/±0.2 rad
	Joint reference offset	±0.1 rad
Robot body	Torso CoM offset (x/y/z)	±0.025/±0.05/±0.05 m
	Encoder bias	±0.01 rad
Contact	Foot friction coefficient	[0.3,1.2]
	Push velocity (every 1–3 s)	±0.5 m/s, ±0.78 rad/s
Observation noise	Projected gravity	±0.05
	Base angular velocity	±0.2 rad/s
	Joint position	±0.01 rad
	Joint velocity	±0.5 rad/s
Tendon execution	Response delay, $\tau_{delay}$	0–2 steps (0–40 ms)
	Equiv. stiffness/damping, $k_{eff},d_{eff}$	×[0.8, 1.2]
	Mapping residual, $\delta_{M}$	±0.05 rad

**Table 3:** PPO training hyperparameters for the Droid X3 tracking task.

Parameter	Value	Parameter	Value
Parallel environments	8192	Discount factor, γ	0.99
Rollout length	24	GAE factor, λ	0.95
Learning epochs	5	Clip range, ϵ	0.2
Mini-batches	4	Value loss coef., $c_{v}$	1.0
Learning rate	$1\times 10^{-3}$ (adaptive)	Entropy coef., $c_{h}$	0.005
Desired KL	0.01	Max gradient norm	1.0
Hidden layers	(1024,1024,512,256)	Activation	ELU
Initial action std	1.0	Control frequency	50 Hz

**Table 4:** Recoverable composition of the processed motion archive. Windows may overlap; duration is computed from non-overlapping source frames. Boldface identifies the aggregate total row.

Source Group	Sequences	Frames	Duration (h)	Windows
LAFAN1	57	608,818	3.382	1386
Pico-record	9	93,951	0.522	213
SEED	63,235	22,945,495	127.475	77,623
TWIST2	31,058	15,833,851	87.966	47,869
**Total**	**94,359**	**39,482,115**	**219.345**	**127,091**

**Table 5:** Randomly selected physical trajectory fragments from repeated trials on two Droid X3 units at 50 Hz. OFF denotes the fixed static joint-to-motor conversion. Under ON, the frozen mapper receives the complete policy-level desired-joint vector and measured plant state and directly outputs the complete motor-position command; no robot-specific static-calibration command is added. Unequal frame counts reflect different visualization boundaries. These rows are illustrative and are not the repeat-level statistical units.

Unit	Motion	Condition	Frames	Duration (s)	Joint MAE (Rad/Joint)	Torso Error (Deg)
1	Walk	OFF	768	15.36	0.1296	5.369
1	Walk	ON	790	15.80	0.1139	5.197
1	Squat	OFF	833	16.66	0.1300	6.657
1	Squat	ON	738	14.76	0.1109	4.261
1	Dance	OFF	1041	20.82	0.1327	6.510
1	Dance	ON	1077	21.54	0.1089	5.000
2	Squat	ON	833	16.66	0.1088	4.538
2	Dance	ON	1135	22.70	0.1109	3.795

**Table 6:** Archived training-run diagnostics. Runs A–C use different motion archives with the same recorded adaptive-sampling parameters. Iterations are measured to reward 20; reward score is mean reward normalized by the common reference peak 36.67, and the episode-length proxy is not a measured hard-motion success rate. Here, ↓ denotes lower-is-better and ↑ denotes higher-is-better. Boldface identifies Archive C, the retained run discussed below; it does not indicate the best value in every column.

Record	Iter. ↓	Reward Score (%) ↑	Episode-Length Proxy (%) ↑	Entropy
Archive A (run 0609)	10,551	52.3	31.2	0.743
Archive B (run 0610)	1412	70.9	61.8	0.709
**Archive C (run 0608)**	**1604**	**78.1**	**72.3**	**0.665**

## Data Availability

The processed archive composition, derived tracking-error records, and reported aggregate diagnostics are described in the article and accompanying project material. Raw robot system-identification trajectories, complete trial manifests, historical dataset-split manifests, and all training checkpoints are not publicly archived with this manuscript. Further inquiries can be directed to the corresponding author.

## Supplementary Materials

The following supporting information can be downloaded at https://www.mdpi.com/article/10.3390/biomimetics11090607/s1, Video S1: Physical whole-body reference-motion tracking and Mapping-OFF/ON comparisons on the Droid X3 humanoid.
